# Acceptability, Preferences, and Palatability of Diets Containing Summer and Winter Brassica Forage in Growing Pigs: A Pilot Study

**DOI:** 10.3390/ani10061080

**Published:** 2020-06-23

**Authors:** Jaime Figueroa, Katalina del Río, Fernanda Romero, Juan Pablo Keim, Mónica Gandarillas

**Affiliations:** 1 Departamento de Ciencias Animales, Facultad de Agronomía e Ingeniería Forestal, Pontificia Universidad Católica de Chile, Macul, Santiago 7820436, Chile; figueroa.jaime@uc.cl; 2 Instituto de Producción Animal, Facultad de Ciencias Agrarias, Universidad Austral de Chile Independencia 631, Valdivia 5110566, Chile; katalina.delrio@alumnos.uach.cl (K.d.R.); fernanda.romero@alumnos.uach.cl (F.R.); juan.keim@uach.cl (J.P.K.)

**Keywords:** feeding behavior, forage rape, growing pigs, kale, summer turnip, swede

## Abstract

**Simple Summary:**

The inclusion of fiber in pigs’ commercial diets may represent an opportunity to reduce feeding costs and benefit animals’ health and welfare. However, antinutritional factors that generate a bitter taste may reduce the voluntary intake of animals. The present experiments evaluated growing pigs’ feeding behavior for winter and summer brassicas, when incorporated on commercial diets as a replacement for wheat middlings. Experiment 1 studied the feeding behavior of pigs when summer turnip or forage rape were included into the diets, while experiment 2 studied the inclusion of kale and swede by replacing 15% of wheat middlings. No differences were found between diets that included brassicas and control diet in pigs’ acceptability or palatability. However, during preference tests of experiment 2 (winter brassicas), diet that incorporated swede presented a higher consumption than control diet and a diet that incorporated kale. This suggests that brassica forage may be incorporated in growing pigs’ diets without negative repercussions in animals’ oral perception during short term feeding tests.

**Abstract:**

Brassica forage may be included in pigs’ diet as a dietary fiber ingredient to reduce feeding costs, benefit gut health, immune system, reproductive traits, and welfare. However, they contain antinutritional factors which may affect feeding behavior. This study evaluated feeding behavior of growing pigs offered winter (kale and swede) and summer (turnip and forage rape) brassicas incorporated on their diets as dried ground meal. Two consecutive experiments with six growing castrated male pigs were conducted. Experiment 1 evaluated the inclusion of turnip bulbs and forage rape, while experiment 2 studied inclusion of kale and swede bulbs. Brassica meal was included at 15% of the diet by replacing wheat middlings (control diet). In each experiment, pigs were offered experimental diets over six consecutive days for 10 min to test their acceptability (day 1–3) and preferences (day 4–6). No differences were found between diets that included brassicas and control diet in pigs’ acceptability or palatability (*p* > 0.05). However, during preference tests of winter brassicas, swede presented a higher consumption than control and kale (*p* < 0.05). This suggest that brassicas may be incorporated in growing pigs’ diets without negative effects in animals’ oral perception during short term feeding tests. Nevertheless, the long-term effects need to be explored.

## 1. Introduction

Historically, ingredients with high dietary fiber (DF) concentrations were not recommended for pig diets when intensively raised, since fiber decreases the digestible energy of the diet, reducing the expression of growth potential of pigs [1,2]. However, the inclusion of DF has been recently re-evaluated due to its benefits in gut health, immune system, reproductive traits, and welfare [3,4]. Also, high DF ingredients represent an opportunity to reduce feeding costs by replacing cereal and oilseed meals with agro-industrial byproducts that have increased in availability, such as those from the bioethanol (DDGS from cereals), the wheat flour milling (wheat middlings, wheat shorts, and wheat bran) and sugar (sugar beet pulp) industries, among others [5]. Wheat middlings and DDGS can be incorporated at 19% and 30% respectively in pigs’ diet as a source of DF, without reducing growth performance [6]. The inclusion level of DF varies depending on animal factors (age and production level) and the characteristics of the feed ingredient itself (fiber type and its physical and chemical properties. Pigs can digest and utilize DF, due to the capacity of their hindgut to absorb microbial fermentation end-products from DF such as short chain fatty acids (SCFA), branched chain fatty acids, lactate, and amines [4]. Brassica forages are annual crops that have been used as a supplement to pastures in times of seasonal shortage [7,8] and when water resources are limited [9]. Summer brassicas include turnip (*Brassica rapa* L. subsp. *rapa*) and forage rape (*B. napus* L. subsp. *biennis*) [7], whereas winter brassicas include kale (*B. oleracea* L. subsp. *acephala*) and swede (*B. napus* L. subsp. *napobrassica*) [8]. The chemical composition of brassicas varies due to the leaf/bulb-stem ratio [10,11]. The crude protein (CP) content of leaves can range from 15–25% on a DM basis, whereas the bulb (root) of turnips and swedes varies from 9% to 16% of the DM [7,12]. In terms of sugar content (raffinose, sucrose, glucose, fructose), swede bulbs are higher (32%) followed by turnip bulbs (21%), whole plant kale (18%), and rape (14%). Whereas starch content varies 6–11% in forage rape, 1–2% in kale leaves, 1–3% in swede bulbs, and 7.6–16.5% in turnip bulbs [11]. In general, brassica crops have a high fiber (soluble and insoluble) content. The insoluble fiber (IF in terms of NDF) ranges from 16.5% to 19.6% in swedes, 18% to 24% for turnip and rape, and 27.1% to 32.8% for kale; whereas soluble fiber (SF) ranges from 24% to 38% [11]. The SF is mainly composed of pectins (7–9%), galactans and β-glucans, among others [7,8,12,13].

In pig diets, the inclusion of brassicas may present opportunities, considering that alternatives to cereal grains have been investigated to reduce feeding costs [14]. Oligo- or polysaccharides containing fructose have been shown to stimulate beneficial hindgut microbiota [15]. Moreover, its fermentable fiber has been shown to reduce nitrogen (N) excretions [16]. Nevertheless, information about the inclusion of brassicas forages in pigs is scarce. Inclusion of swedes as a partial replacement for barley (20–40%) in traditional intensive systems has shown to reduce growing rates in fattening animals [17,18]. However, the incorporation of swedes as an extra food offered directly at ground level (in semi-extensive systems) significantly reduced the time that sows spend rooting and also increased their gut fill perception [19]. There are some studies using other brassica species but as byproducts from the oilseed industry [20,21,22], thus may not be applicable to compare with the vegetative (forage) species. 

The potential inclusion of brassicas in commercial diets will additionally depend on their hedonic proprieties that will determine the voluntary intake of animals [23]. To the best of our knowledge, neither short term acceptability (total amount consumed when only one consumption option is present), preference (election of one diet over another), nor palatability (pleasure perception) studies in growing pigs diets containing forage brassicas have been reported. Since brassicas are relatively high in sugar concentration compared to other DF sources, pigs may be attracted to eat them compared with typical DF ingredients such as wheat middlings. Therefore, the objective of the present work was to evaluate growing pigs feeding behavior for winter and summer brassicas incorporated in their diets as a replacement for wheat middlings.

## 2. Materials and Methods

Experiments were conducted at the non-ruminant experimental facilities of Universidad Austral de Chile’s research station, Valdivia, Chile. All the procedures, including animal care and handling procedures, followed national legislation (Law No. 20,380 on Protection of Animals; Decree No. 29 about regulation on the protection of animals during their industrial production, their commercialization and in other areas to hold animals), whose application is supervised by the National Service of Agriculture and Livestock (SAG), the competent authority in this matter.

### 2.1. Forage Production and Collection

Turnips (4 kg seed/ha) and forage rape (5 kg/ha) were established in October 2017, whereas kale (5 kg/ha) and swedes (3 kg/ha) were sown in November 2017. Prior to establishment, soil analyses were conducted (20 cm depth) and 3000 kg/ha of limestone (91% CaCO_3_) were incorporated into the soil. A fine, well-compacted seed bed was prepared, and seeds incorporated in the paddocks. At sowing, fertilizer (600 kg/ha of NPK ratio 5:20:20) was applied to correct the soil nutrient deficiencies in order to supply the nutrient demands of crops. After emergence, weeds were controlled chemically by applying Lontrel 3A (clopyralid 475 g of active ingredient (a.i.)/L) and Tordon 24 k (picloram 240 g a.i./L) at doses of 300 and 200 cc/ha, respectively. Urea was applied (200 kg/ha 46% N) once brassica plants reached three leaves. Summer and winter brassicas were harvested in the morning between 05:00 a.m. and 07:00 a.m. at 100 and 190 days after plant emergence, respectively. All plants were in a vegetative stage of growth at harvest. Summer turnip and swede were collected manually, leaves separated from bulbs and soil attached to the bulbs was removed. Rape and kale were harvested with a cutter-bar mower (model 140; Bertolini, Reggio Emilia, Italy) at respective heights of 5 cm and 20 cm above ground level. For the feeding trial, only bulbs were used for summer turnip and swede and, whole plant for kale and rape. Forage samples were immediately carried to the Animal Nutrition Laboratory of the Universidad Austral de Chile, where they were chopped in small pieces of 1 cm^2^ and weighed in green, leaving them for 48 h at 60 °C in a drying oven. After 48 h, they were weighed and ground by passing them through a 5-mm sieve and then stored in plastic sealed bags. Experiments of feeding behavior in pigs were carried out from December 2018 to January 2019.

### 2.2. Feedstuff Chemical Analyses

Before the start of experiments, ground brassicas were analyzed for dry matter (DM), crude fiber (CF), ether extract, ash and acid detergent fiber (ADF) according to [24] (procedures 978.10, 942.05, 920.39, and 973.18 respectively), gross energy using an adiabatic calorimetry [24] and NDF using a heat stable amylase [25]. Nitrogen content was measured by combustion (Model FP-428 Nitrogen Determinator; LECO, St. Joseph, MI, USA) and was used to calculate CP content (*N* × 6.25).

### 2.3. Animals and Housing

Six castrated male pigs [(PB 337 × Camborough) PIC genetics] of 25.2 ± 1.1 kg and 70 days old were purchased from a commercial company and transported to the experimental facilities. Animals were identified with plastic ear tags and allocated in individual pens (1.7 m × 0.85 m) inside a controlled room of concrete floor. Pigs were housed under controlled temperature conditions (20 ± 2 °C) and ventilation were set through the day to remove excess of humidity and stale air. A corn–soybean powder commercial feed, formulated to meet their nutritional and energy requirements, was provided ad libitum by using pan feeders [26] except for 1 h before and after each test. Fresh water was also provided ad libitum through a stainless-steel nipple. No environmental enrichment was delivered to animals. Before the onset of the experimental period, pigs were acclimated for 1 week to facilities and management conditions.

### 2.4. Experimental Procedures

Five diets were formulated to perform two consecutive experiments. The feed ingredients and the nutritional composition of diets are reported in Table 1. Experiment 1 studied the feeding behavior of pigs when summer brassicas (turnip bulbs and whole plant forage rape) were included into pigs diets, while experiment 2 studied the feeding behavior of pigs when winter brassica crops (whole plant kale and swede bulbs) were included. In both experiments, brassica crops were offered as ground dried meal and included at 15% of the diet, replacing wheat middlings which were included in the control diet. Experimental diets were formulated to meet the nutritional requirements of growing pigs [26] and were iso-energetic, iso-nitrogenous, and iso-fibrous. Using more that 15% of brassica crops and wheat middlings would not fulfil net and metabolizable energy for growing pigs. 

#### 2.4.1. Experiment 1, Summer Brassica Crops

After the acclimatization week, pigs were offered during six consecutive days experimental diets during 10 min in the morning (10:00 a.m.) to test their acceptability (day 1–3) and preferences (day 4–6). Experimental diets included: (i) Control diet: 85% basal diet + 15% wheat middling; (ii) forage rape (FR) diet: 85% basal diet + 15% dried ground forage rape; (iii) Turnip diet: 85% basal diet + 15% dried ground turnip bulbs (Table 1). In the acceptability test, one pan feeder was placed at the front of each pen and the consumption of each diet (1 diet per day) was estimated by the difference between the initial and final weight of the feeder. The order of delivered diets were counterbalanced between animals. In this way, two pigs on day 1 received the control diet, two pigs received the FR diet, and two pigs received the turnip diet. On days 2 and 3, animals received the remaining diets that also were counterbalanced between animals. Video cameras (four video cameras, IR outdoor cameras 700tvl 1/3 cmos Sony, SENKO SA, Santiago, Chile) were placed in the front of each pen to allow behavioral recording of each animal over the experimental sessions. Consumption time (time eating at the pan; CT) and approaches (number of times the pan was approached with a consumption result; A) were assessed from the video recordings by focal continuous sampling over the 10 min test period. Palatability was estimated through consumption patterns (CT/A) [23,28]. Diets offered were counterbalanced between pigs and days to avoid possible order bias. 

After the acceptability test, pigs were tested for three consecutive days to study their preference among the three dietary treatments. Preference was studied in a two-feeder test where two equidistant pan-feeders were placed in the front of each pen during 10 min. All possible combinations were tested (FR vs. Control; Turnip vs. Control; and FR vs. Turnip) and the order of comparisons were counterbalanced between pigs and days to avoid possible order bias. Moreover, the right-left position of diets was also counterbalanced between animals in each diet comparison to avoid possible side bias. Consumption of each option during the preference test was estimated by the difference between the initial and final weight of the feeder.

#### 2.4.2. Experiment 2, Winter Brassica Crops

After a three-week break, pigs (45 kg; 100 days old) were offered experimental diets containing the winter brassicas over six consecutive days for 10 min in the morning (10:00 a.m.) to test their acceptability (day 1–3) and preferences (day 4–6) for the diets. Experimental diets included (i) Control diet: 85% basal diet + 15% wheat middling; (ii) Kale diet: 85% basal diet + 15% dried ground kale; (iii) Swede diet: 85% basal diet + 15% dried ground swede bulbs (Table 1). Experimental procedures to measure animals’ acceptability, palatability, and preferences were the same as described for experiment 1. 

### 2.5. Statistical Analysis

Pigs were randomly allocated to three dietary treatments in a replicated 3 × 3 Latin square design. The consumption by pigs during the 10-min acceptability and preference tests were analyzed using the MIXED procedure of the statistical package SAS (9.4; SAS Inst. Inc.; Cary, NC, USA), taking into account the diet consumed (FR, Turnip, Control for Exp. 1 or Swede, Kale and Control for Exp. 2) as the main factor, the random effect of the square; the random effect of pig nested within square; and the fixed effect of the period. For the choice test, the experimental unit (pig) was considered a random effect specifying the covariance structure of the residual matrix as completely general (unstructured). 

Consumption pattern was analyzed with ANOVA using statistical package SAS with the experimental diet as the main factor. Although the primary analysis was over the whole testing period, we also performed indicative analyses at 0–5 min and 6–10 min to explore the distribution of possible different effects. Mean values are presented as least square means, and significance assessed at a criterion of 5%.

## 3. Results

### 3.1. Experiment 1, Summer Brassicas 

**Figure 1:**
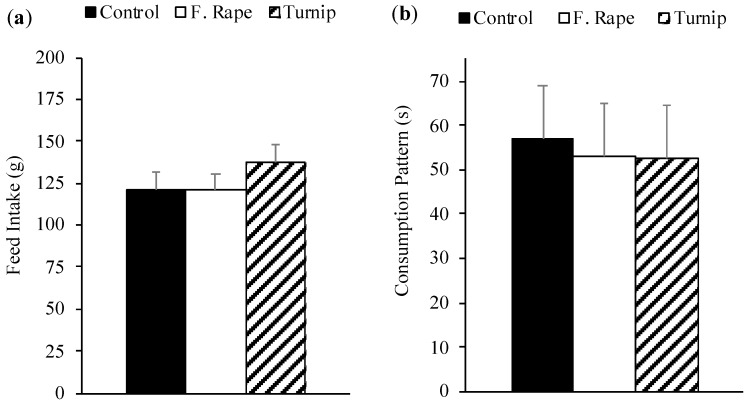
Pigs’ feed intake (**a**) and palatability (**b**) expressed as consumption patterns (consumption time/number of approaches to the feeder) of corn–soybean base diets (least squares means + standard error) with the inclusion of 15% of forage rape, turnip, or wheat middlings (control) when animals were offered one consumption option for 10 min.

No differences were found between experimental diets in pigs’ acceptability (*p* = 0.453) or palatability (*p* = 0.962; Figure 1). The analysis of the consumption pattern by periods is shown in Table 2. Dietary treatments had no effect in either the first (0–5 min) or second period (6–10 min) of the test on pigs’ consumption patterns. 

**Figure 2:**
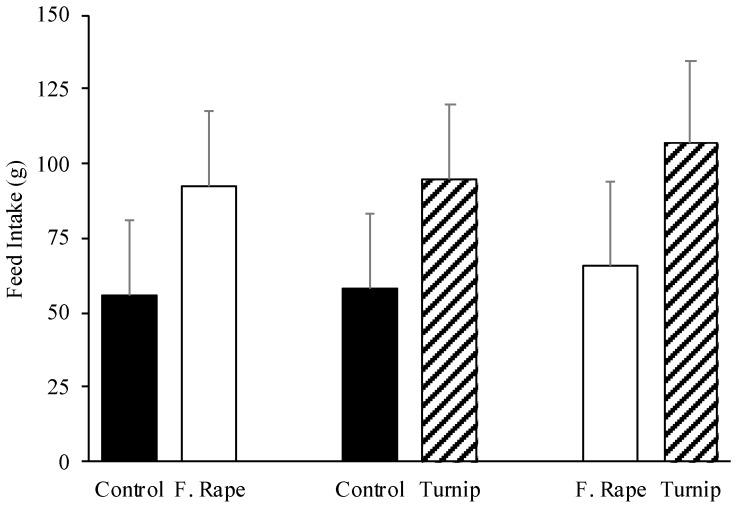
Pigs’ feed intake (g) of corn–soybean base diets (least squares means + standard error) with the inclusion of 15% of forage rape, turnip, or wheat middlings (control) when animals were offered a two-feeder preference test during 10 min.

Results of the two-choice preference test are shown in Figure 2. No differences were observed between the consumption of control vs. FR (*p =* 0.324), control vs. rurnip (*p =* 0.334), or FR vs. turnip (*p =* 0.325). Although the results in the preference tests did not show significance, it is observed numerically that the animals consumed less control diet (about half) when exposed simultaneously with diets to which turnip or FR were incorporated. In the direct comparison between the diets with Turnip and FR, the animals’ consumption was numerically greater for turnip.

### 3.2. Experiment 2, Winter Brassicas

**Figure 3:**
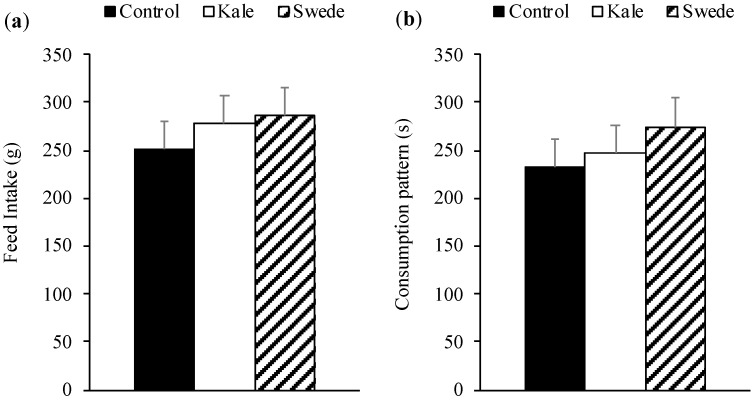
Least squares means ( + Standard Error) of pigs feed intake (**a**) and palatability expressed as consumption patterns; consumption time/number of approaches to the feeder (**b**) of corn–soybean base diets with the inclusion of 15% of kale, swede, or wheat middlings (control) when animals were offered one consumption option during 10 min.

As in experiment 1, no differences were found between the three diets in pigs’ acceptability (*p* = 0.741) or palatability (*p* = 0.949; Figure 3). The analysis for consumption pattern by periods is shown in Table 3. Dietary treatments had no effect in either the first (0–5 min.) or second period (6–10 min.) of the test on pigs consumption patterns (*p* > 0.05).

**Figure 4:**
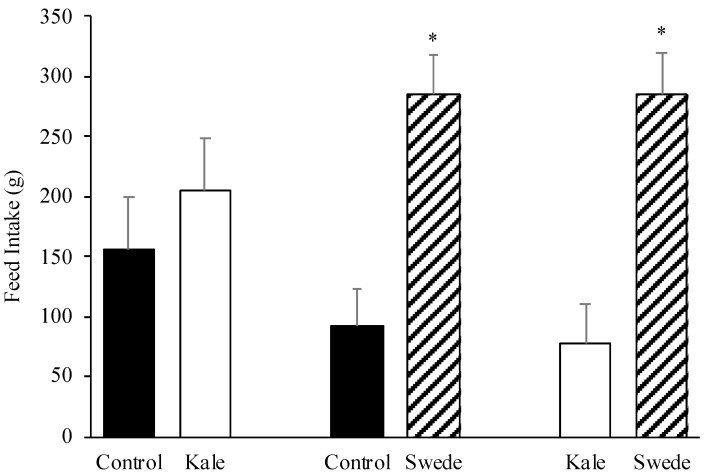
Least squares means (+ standard error) of pigs’ feed intake (g) of corn–soybean base diets with the inclusion of 15% of kale, swede, or wheat middlings (control diet) when animals were offered a two-feeder preference test during 10 min. * *p* < 0.05.

Results of the two-choice preference test are shown in Figure 4. No differences were observed between the consumption of control vs. kale (*p* = 0.491), nevertheless the diet that incorporated swede presented a higher consumption than control (*p* = 0.003) and kale (*p* = 0.004).

## 4. Discussion

Animal perception for sweet or bitter flavors may influence the acceptability for a new ingredient and pigs are able to distinguish between them, having a natural or innate preference for sweet flavors [29] (as they are related to high energy ingredients) and an innate aversion for bitter flavors (which may be associated to toxins) [30]. The inclusion of brassicas in pig industry as an alternative to traditional cereals may reduce feeding costs [14]. However, they often present antinutritional factors (ANF) (i.e., glucosinolates) which confer some bitter taste and specific odors as part of a defense mechanism of the plant to depredators [21]. The two experiments explored growing pigs’ feeding behavior, expressed as acceptability, preferences, and palatability for summer and winter brassicas. We observed that animals did not present differences between summer brassicas and control diet. When winter brassicas were tested, swede was preferred over the control and kale diets, without differences in terms of acceptability and palatability. It is worth mentioning that this is a pilot study with brassicas was offered as dried meals. In outdoor feeding systems, brassicas are offered as whole plants without processing, thus preference and acceptability of pigs for fresh brassicas may differ from these results, as factors such as the water content and bulkiness of feedstuffs and, the way forages are offered (grazed whole plant v/s dried meal) may affect preference and acceptability of pigs [30]. 

If a given feedstuff is showed for a first time to a pig, the animal could develop neophobic conducts (fear to a new ingredient) [31]. The previous experience of animals with an ingredient through repeated exposure or previous associations of the ingredient with positive or negative post-ingestive consequences may change the behavior of animals when that ingredient is included in future commercial feeds [32]. Therefore, the reaction of pigs in front of diets is a complex process determined for the chemical, physical, and nutritional properties of a given feedstuff, but also by the internal status of the animals and their previous experience [30]. Sweetness and bitterness affect pigs feeding behavior [33]. Glucosinolates concentration in brassicas vary within species, organ, and phenological stages [34]. As an example, the glucosinolate content in swedes is greater than in kale [7]. However, this ANF may severely decrease when are exposed to thermic treatments [35], while cooking can decrease up to 50% of glucosinolates, and a post-harvest drying from 20% to 50% [36,37]. In these experiments, both winter and summer brassicas were oven-dried at 60 ºC for 48 h, thus ANF concentrations may have decreased and therefore the bitter tastes might have been reduced in all brassicas. This could explain that pigs did not present innate aversion, as it was reflected in similar intakes to the control diet or even a higher preference when swede was included. 

Diets used in this experiment had the same ingredients and the source of variation was the fiber ingredient: wheat middlings in the control diet and specific summer or winter brassicas as experimental diets. Moreover, diets were formulated to be iso-energetic, iso-nitrogenous, and iso-fibrous. In experiment 1, the three ingredients were different in the type of fiber (SF and IF) and non-structural carbohydrates. Wheat middlings had more NDF content (34.97%) compared to bulb turnip (17.9%) and forage rape meals (18.6%), more starch (21.8%, 11.1%, and 15.9%, for wheat middlings, bulb turnip and forage rape meals, respectively) and less sugars (0%, 20.8%, and 14.6% for wheat middlings, bulb turnip, and forage rape meals, respectively). In experiment 2, wheat middlings (34.97%) and kale meal (33.4%) had considerably more NDF content compared to bulb swede meal (14.7%), whereas sugar content among is greater for bulb swede meal (37.0% of sugars, with 20.7% glucose and 14.6% fructose), followed by kale meal (16.1% of sugars, with 7.8% sucrose, 4.3% glucose, and 3.4% fructose) and wheat middlings without sugar concentration [11,26]. We expected to find differences in acceptability or preference among diets since pigs show a marked attraction for sweet ingredients [38] and thus, an increased consumption in short tests. However, in the acceptability test we found no differences among diets, so these new ingredients were acceptable in the same way as wheat middlings, regardless of their greater sugar content. These pigs were raised eating a diet based on a corn and soybean meal, so they had no previous experience with any of the three new ingredients. This situation probably might denote neophobic reaction in animals, explaining no differences found in pigs feeding behavior.

Regarding the preference test, the diet with 15% of swede bulb was preferred over the diets with wheat middlings and kale. Swede have a greater concentration of glucose and fructose as compared to kale [11]. The sugar content may affect preferences creating a higher hedonic reaction in rats and pigs [23,28]. Swede presented the highest sugar concentration among all brassicas tested in both experiments, however it has been reported that sugar concentration in swede bulbs varies among varieties from 29% to 36% of total DM, as well as glucose and fructose concentrations [11]. This variation in sugar concentration needs to be considered as it may affect preference of growing pigs. As the preference test was performed after acceptability test, pigs already new (in some degree) diets that incorporated brassicas when were exposed to two-feeder preference test, decreasing a possible effect of neophobia, making it possible to observe marked preferences. The lack of differences among the kale and control diet may be explained because the sugar content of kale was not high enough to make a difference with respect to the wheat middling diet. Even though sucrose was the main sugar in kale and, it has been reported that sucrose and fructose are preferred over glucose in pigs [29]. Furthermore, nitrate concentrations are greater in kales than in other brassicas [7]. Nitrate can convert hemoglobin to methaemoglobin, causing anoxia, and therefore may reduce intake [39]. In forages with a high proportion of stem in pig diets, such as kale, the level of inclusion of a high fiber ingredient affects preference. For example, dehydrated alfalfa (*Medicago sativa*) and sugar beet pulp (*Beta vulgaris*) are less preferred by pigs than wheat bran when the inclusion level was 13% [40].

Despite the importance of pleasure perception in pig’s intake of commercial diets, feed palatability has typically been inferred indirectly from measurements of preference or acceptance [30], providing weak evidence of pigs’ hedonism because they are influenced by factors beyond palatability such as satiety or hunger [41,42]. Just a few experiments have directly explored pigs’ hedonic perception in front of ingredients or additives [23,28,30]. Recently, palatability has been studied in pigs through the analysis of consumption patterns [23], analogous to the lick cluster size analysis in rats [42,43], where a relationship between the consumption time and the number of approaches to the site of consumption is assessed. However, no studies have been performed until today regarding the perceived palatability of forage brassicas in pigs. Present results showed no differences between brassicas and wheat middlings in terms of palatability. As we explained previously, the reduction of ANF may decrease bitter taste perception due to a previous thermic treatment increasing pigs’ preferences, but also the hedonic perception of feed to sweet sugars presented on the product. Consumption patterns in pigs are directly related to sucrose inclusion in water solutions observing more hedonic reaction in the sweetest solutions as in rats [23]. However, as we explained before, it is probably that neophobia in front of brassica did not allow us to observe clear differences between diets.

## 5. Conclusions

Results of the present experiments suggest that forage brassicas may be incorporated in growing pigs’ diets without negative repercussions in animals’ oral perception during short term feeding tests. Nevertheless, the long-term effects of brassicas incorporation in pigs’ diets on their performance needs to be explored.

## Figures and Tables

**Table 1:** Ingredients and chemical composition of experimental diets tested in growing pigs. Brassica crop meal was included at 15% of the diet by replacing wheat middlings that was included in the control diet.

	Control	Turnip	Forage Rape	Swedes	Kale
**Ingredients**					
Corn (%)	42.1	42.1	42.1	42.1	42.1
Soybean meal (%)	33.5	33.5	33.5	33.5	33.5
Soybean oil (%)	5.5	5.5	5.5	5.5	5.5
Wheat middlings (%)	15	0	0	0	0
Turnip meal (%)	0	15	0	0	0
Forage rape meal (%)	0	0	15	0	0
Swede meal (%)	0	0	0	15	0
Kale meal (%)	0	0	0	0	15
Calcium carbonate (%)	0.4	0.4	0.4	0.4	0.4
Salt (%)	0.35	0.35	0.35	0.35	0.35
Calcium biphosphate (%)	1.15	1.15	1.15	1.15	1.15
Premix vit-min (%)	1.5	1.5	1.5	1.5	1.5
Celite (%)	0.5	0.5	0.5	0.5	0.5
Overall (%)	100	100	100	100	100
**Nutrient Concentration**					
Dry matter (%)	88.4	89.29	89.23	88.39	89.88
Ash (%)	6.68	8.17	7.04	7.73	8.44
Crude protein (%)	20.93	20.91	20.58	22.57	20.71
Ether extract (%)	8.21	7.5	7.72	8.78	7.79
Crude fiber (%)	3.96	2.49	2.49	4.54	4.53
NDF (%)	13.16	10.46	10.72	12.6	10.7
ADF (%)	4.91	5.15	5.51	6.12	5.3
Starch (%) ^1^	31.14	29.76	28.27	31.25	25.7
Sugars (%) ^1^	4.38	7.67	3.97	8.98	8.6
GE (kcal/kg) ^1^	4123	4122	4117	3985	4124
DE (kcal/kg) ^1^	3442	3559	3543	3444	3511
ME (kcal/kg) ^1^	3284	3401	3384	3266	3349
NE (kcal/kg) ^1^	2502	2521	2511	2418	2467

^1^ values were predicted using Evapig^®^ software [27]; NDF (neutral detergent fiber); ADF (acid detergent fiber); GE (gross energy); DE (digestible energy); ME (metabolizable energy); NE (net energy).

**Table 2:** Consumption pattern (consumption time/number of approaches to the feeder) over 10 min from growing pigs (*n* = 4) exposed to corn–soybean base diets with the inclusion of 15% of forage rape, turnip, or wheat middling as the control diet expressed by the first (0–5 min), last (6–10 min), and total (0–10 min) periods of the test.

Consumption Pattern (CT/A)	Control	F. Rape	Turnip	SEM	*p*-Value
0–5 min	95.0	57.2	55.7	38.6	0.574
6–10 min	52.2	50.8	49.3	27.9	0.979
0–10 min	57.0	53.2	52.8	58.7	0.962

**Table 3:** Consumption pattern (CT/A) over 10 min from growing pigs (*n* = 4) exposed to corn–soybean base diets with the inclusion of 15% of kale, swede, or wheat middlings as the control diet expressed by the first (0–5 min), last (6–10 min) and total (0–10 min) period of the test.

Consumption Pattern (CT/A)	Control	Kale	Swede	SEM	*p*-Value
0–5 min	186.5	145.1	197.6	46.4	0.707
6–10 min	126.7	186.0	154.3	47.6	0.684
0–10 min	233.0	247.0	274.6	92.6	0.949
